# The prognostic values of serum markers in hepatocellular carcinoma after invasive therapies based on real‐world data

**DOI:** 10.1002/jcla.23932

**Published:** 2021-08-17

**Authors:** Bo Li, Aixia Liu, Yi Wen, Guang Yang, Jing Zhao, Xiaohan Li, Yuanli Mao, Boan Li

**Affiliations:** ^1^ Department of Clinical Laboratory The Fifth Medical Center of Chinese PLA General Hospital Beijing China

**Keywords:** hepatocellular carcinoma, overall survival, real‐world data, recurrence‐free survival, tumor biomarkers

## Abstract

**Background and aims:**

Hepatocellular carcinoma (HCC) is one of the most common malignancy with poor prognosis, and the mortality rate remains high. More than 70% of HCC patients have recurrence within 5 years after treatment. The purpose of this study is to evaluate the prognostic values of serum markers with retrospective data.

**Methods:**

We applied real‐world data (RWD) to analyze the prognostic values of six serum markers for HCC patients after treatment, including α‐fetoprotein (AFP), α‐fetoprotein‐L3 (AFP‐L3), Golgi protein73 (GP73), alanine aminotransferase (ALT), albumin (ALB), and total bilirubin (TBil). A total of 268 cases were enrolled to analyze recurrence‐free survival (RFS), and 104 cases were used to analyze overall survival (OS).

**Results:**

Our results demonstrated that patients with higher AFP and AFP‐L3 had shorter RFS (*p *= 0.016 and 0.004), while higher GP73, ALT, and TBil experienced longer RFS (*p* = 0.000, 0.020, and 0.019). Patients with high‐level GP73, ALT, TBil, and low‐level ALB had significantly higher mortality rate (*p*=0.035, 0.008, 0.010, and 0.005). Multivariate analysis revealed that GP73 (HR = 1.548, *p* = 0.001) and ALT (HR = 1.316, *p* = 0.046) were identified as independent prognostic factors for RFS, ALB (HR = 0.127, *p* = 0.007), and ALT (HR = 0.237, *p* = 0.01) were identified as independent prognostic factors for OS. Subgroups analysis showed that GP73 had better prognostic values than other serum markers in early‐stage HCC (*p* = 0.023).

**Conclusions:**

Our study demonstrates that AFP, AFP‐L3, and GP73 can be used as prognostic indicators for predicting the recurrence of HCC, while liver function tests have better survival prediction values. GP73 can act as a promising prognostic marker for early‐stage HCC.

## Graphical abstract

Prior to nasotracheal intubation (NTI), topical nasal vasoconstrictors are used to prevent NTI‐related epistaxis (NTIRE). Our study shows that well‐lubricated nasotracheal intubation does not require pretreatment with ephedrine to reduce NTIRE.

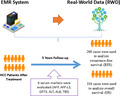

## 1 INTRODUCTION

Hepatocellular carcinoma (HCC) is one of the most common malignant tumors worldwide. In 2018, A Cancer Journal for Clinicians had published the latest global cancer report which revealed that the morbidity and mortality of liver cancer were sixth and fourth, respectively, in all malignant tumors. Approximately 800,000 patients develop liver cancer and 780,000 patients die of it in the world each year. Among these liver cancer patients, 75%–85% of them are HCC cases.^1^ HCC also has high death rate and was difficult to be cured, and the median survival time of HCC population was less than 10 months and overall 5‐year survival rate less than 20%.^2^

At present, several therapeutic methods are available to treat HCC, including surgical resection, interventional therapy, cryoablation, and radiofrequency ablation, etc.^3^ However, no matter what methods are applied, recurrence will happen after treatment and leading to treatment failure. Previous studies have reported that more than 70% of patients who underwent invasive therapies had HCC recurrence within 5 years, and most recurrences occurred because of dissemination of primary tumor.^4^ Therefore, the therapeutic effect of HCC is still far from optimistic currently. HCC recurrence is a main obstruction for the improvement in long‐term survival rate of HCC patients.

Various prognostic biomarkers have been developed during the past decades to predict the clinical outcomes of HCC patients.^5^ American Association for the Study of Liver Diseases (AASLD) once recommended imaging techniques, and α‐fetoprotein (AFP) test were used as monitoring methods for HCC recurrence. However, imaging techniques are costly and time‐consuming. AASLD guideline indicated that the re‐elevation of serum level of AFP after treatment is associated with recurrence and poor outcomes.^6^ Compared with imaging techniques, serum marker detection is more rapid and cost‐effective. Except for AFP, other biomarkers such as α‐fetoprotein‐L3 (AFP‐L3) and Golgi protein73 (GP73) are also regarded as potential prognostic biomarkers and are applied broadly at present.^7^, ^8^, ^9^, ^10^ In addition, liver function tests, including alanine aminotransferase (ALT), albumin (ALB), and total bilirubin (TBil), have also shown prognostic values in clinical practice.

However, large‐scaled population‐based investigations about prognostic biomarkers in HCC are absent till now due to difficult longer‐term follow‐up. Hence, in this study we applied real‐world data (RWD) to analyze the prognostic values of serum markers in HCC. RDW is defined as data relating to patient health status and/or the delivery of health care routinely collected from a variety of sources. All the data collected in this study were derived from the electronic medical record (EMR). Compared with trial data, RDW not only can evaluate the clinical values in a more realistic way but also can avoid longer‐term follow‐up process.

## 2 MATERIALS AND METHODS

### 2.1 Subjects

We searched for potentially eligible patients using the EMR system of our hospital from January 2013 to January 2019. All included subjects were HCC patients with invasive therapies, and the medical records were tracked for five years subsequently. The data were collected for further analysis if the definite outcomes (recurrence or death) were acquired. The HCC diagnosis was confirmed by MRI based on guidelines from ministry of health of the People's Republic of China^11^ and guideline from the Chinese Society of Hepatology and the Chinese Society of Infectious Diseases.^12^, ^13^

### 2.2 Laboratory tests

Six routine serum markers were selected for further analysis, including three tumor biomarkers (AFP, AFP‐L3, and GP73) and three liver function tests (ALB, ALT, and TBil). These markers were compared between patients with abnormal serum levels and those with normal serum levels. The judgment criteria are as follows: AFP>25 ng/mL, AFP‐L3>1.0 ng/ml, GP73>150 ng/ml, ALB<35 g/L, TBil>19 μmol/L, ALT>40 U/L. Liver function tests were detected by an automatic biochemical analyzer (AU5800, Beckman Coulter). AFP and AFP‐L3 were measured by Automated Immunoassay Analyzer (COBAS6000, ROCHE). Enzyme‐linked immuno‐absorbent assay kits for GP73 were obtained from Hotgen Biotech. All results collected were measured within one week before treatments.

### 2.3 Statistical analysis

All statistical analyses were performed using SPSS 14.0 software (SPSS, Inc.). Recurrence‐free survival (RFS) was calculated by patients with definite recurrent records, and overall survival (OS) was calculated by patients with definite survival records. Survival curves were plotted by Kaplan‐Meier method, and comparison between groups was analyzed by log‐rank test. The Cox proportional hazard regression model was used for univariate and multivariate analyses. A value of *p *< 0.05 was considered to be statistically significant.

## 3 RESULTS

### 3.1 Clinical characteristics of included patients

**FIGURE 1:**
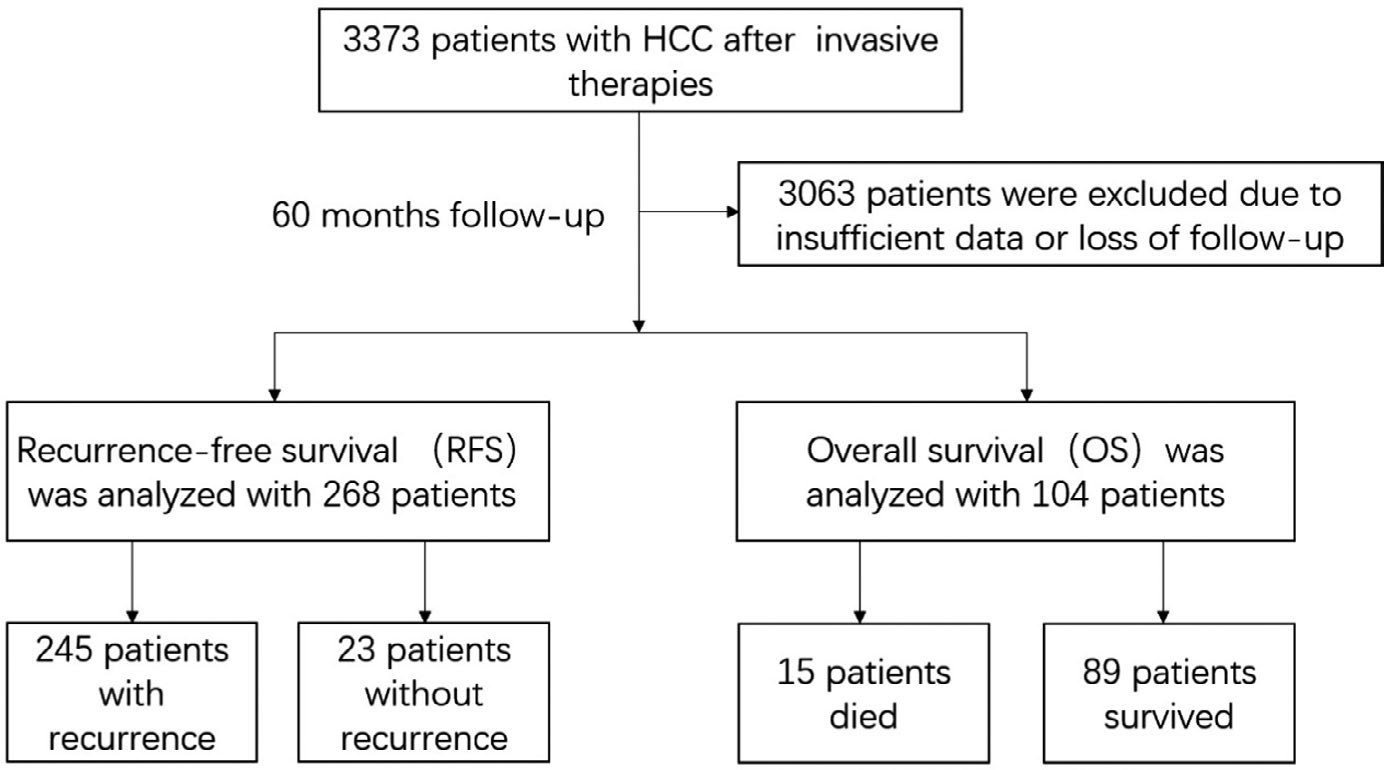
Flowchart of this study

Finally, a total of 3373 HCC patients were checked and 3063 of them were excluded for insufficient data or loss of follow‐up. Among these collected patients, 268 cases had definite recurrent records (245 with recurrence, 23 without recurrence), 104 cases had definite survival records (15 died, 89 survived), and the search workflow is shown in Figure 1. Of the entire study population, 234 were males and 76 were females. The median age was 56 years (range 33–81 years). Seventy‐three cases (23.5%) were suffered from hypertension. Sixty‐five were diabetes mellitus patients (21.0%). A total of 240 (77.4%) were infected by HBV, and 62 cases (20.0%) were infected by HCV. Forty‐three (13.9%) of them had alcohol intake history.

### 3.2 Comparison of survival time in RFS and OS

Log‐rank test was performed with recurrent records which demonstrated that patients with higher AFP and AFP‐L3 had shorter RFS (*p* = 0.016 and 0.004), while those who with higher GP73, ALT, and TBil experienced longer RFS (*p* = 0.000,0.020 and 0.019). The median RFS was only 9 months in high‐level AFP and AFP‐L3 patients. However, in low‐level patients, the median RFS was 13 and 14 months, respectively. Patients with higher GP73, TBil, and ALT had longer RFS, while those with low level had shorter RFS. As regards ALB, there was no significant difference between the abnormal and normal level groups in RFS (*p* = 0.656).

**FIGURE 2:**
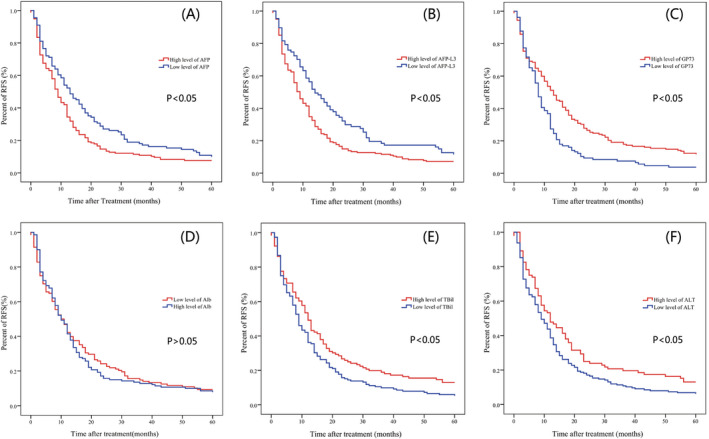
Kaplan‐Meier survival curves for recurrence‐free survival (RFS) in HCC patients. (A and B) RFS was significantly better in low‐level AFP and AFP‐L3 groups than high‐level groups (*p *< 0.05). (C–F) RFS was significantly better in high‐level GP73, TBil, and ALT groups than low‐level groups (*p *< 0.05)

**FIGURE 3:**
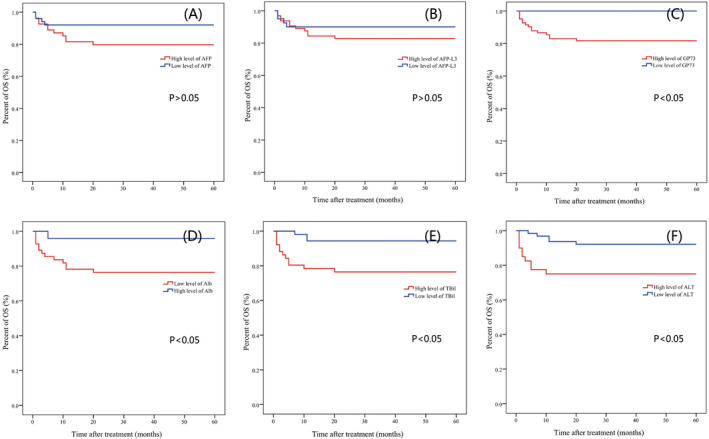
Kaplan‐Meier survival curves for overall survival (OS) in HCC patients. (C–F) OS was significantly better in low‐level GP73, TBil, and ALT groups than high‐level groups (*p *< 0.05). (D) OS was significantly better in high‐level ALB groups than low‐level groups (*p *< 0.05)

Analysis with survival records also revealed that patients with higher GP73, ALT, TBil, and low‐level ALB had significantly higher mortality rate (*p* = 0.035, 0.010, 0.008, and 0.005). The average survival times were 50, 47, 47, and 46 months in abnormal groups and were 60, 58, 57, and 56 months in normal group. In addition, all the patients with low‐level GP73 (≤150 ng/mL) survived until the end of 5 years follow‐up. However, the levels of two tumor biomarkers (AFP and AFP‐L3) were not associated with the mortality of HCC patients (*p *> 0.05). See Table 1, Figure 2, and Figure 3.

**TABLE 1:** Log‐rank analysis for RFS and OS

Variables	RFS				OS			
	Median	Std.error	95% CI	*p*	Means	Std.error	95% CI	*p*
AFP(ng/mL)								
>25	9	0.815	7.402–10.598	0.016	49	2.935	43.415–54.919	0.085
≤25	13	1.755	9.560–16.440		55	2.216	51.036–59.724	
AFP‐L3(ng/mL)								
>1.0	9	0.874	7.286–10.714	0.004	51	2.524	45.911–55.807	0.345
≤1.0	14	2.120	9.845–18.155		54	2.740	48.854–59.596	
GP73(ng/mL)								
>150	13	1.590	9.884–16.16	0.000	50	2.349	45.373–54.724	0.035
≤150	8	0.642	6.741–9.259		60	/	/	
ALB(g/L)								
<35	10	1.331	7.391–12.609	0.656	47	3.133	41.024–53.304	0.005
≥35	10	1.029	7.984–12.016		58	1.555	54.708–60.802	
TBil(μmol/L)								
>19	12	0.950	10.138–13.862	0.020	47	3.311	40.471–53.451	0.008
≤19	9	0.838	7.358–10.642		57	1.599	54.017–60.285	
ALT(U/L)								
>40	12	1.996	8.087–15.913	0.019	46	3.902	38.128–53.422	0.010
≤40	9	1.061	6.920–11.080		56	1.668	52.872–59.410	

### 3.3 Univariate analysis and multivariate analysis for RFS and OS

Six serum markers and gender, age, tumor size, differentiation degree, metastasis, and therapeutic methods were defined as variables. A univariate analysis was conducted first, and those variables with a *p *< 0.05 were included in a multivariate analysis. In univariate analyses, low level of AFP (HR = 0.738, *p* = 0.02) and AFP‐L3 (HR = 0.681, *p* = 0.006) and high level of GP73 (HR = 1.581, *p* = 0.001), ALT (HR = 1.361, *p* = 0.024), and TBil (HR = 1.341, *p* = 0.025) were significantly associated with longer RFS. Tumor size (HR = 0.271, *p* = 0.013), ALB (HR = 0.156, *p* = 0.014), ALT (HR = 0.271, *p* = 0.017), and TBil (HR = 0.213, *p* = 0.016) also showed significance with OS.

In multivariate analyses, the following two independent factors were identified for RFS, they were GP73 (HR = 1.548, *p* = 0.001) and ALT (HR = 1.316, *p* = 0.046). Tumor size (HR = 0.315, *p* = 0.032), ALB (HR = 0.127, *p* = 0.007), and ALT (HR = 0.237, *p* = 0.01) still were identified as independent prognostic factors for OS, see Table 2 and Table 3.

**TABLE 2:** Univariate analysis and multivariate analysis for RFS

Variables	Univariate analysis			Multivariate analysis		
	HR	95% CI	*p*	HR	95% CI	*p*
Gender						
Male	1	0.697–1.257	0.659			
Female	0.936					
Age (y)						
>56	1	0.691–1.142	0.356			
≤56	0.888					
Tumor Size(cm)						
>3	1	0.699–1.161	0.420			
≤3	0.901					
Metastasis						
No	1	0.934–1.710	0.129			
Yes	1.264					
Differentiation						
Low	1	0.888–1.598	0.244			
High	1.191					
Therapeutic Methods						
Resection	1					
Interventional therapy	1.335	0.475–3.747	0.584			
Cryoablation	0.940	0.345–2.560	0.904			
Radiofrequency ablation	1.337	0.480–3.720	0.578			
Comprehensive therapy	0.785	0.278–2.213	0.647			
AFP(ng/mL)						
>25	1	0.571–0.954	0.02	1	0.648–1.613	0.924
≤25	0.738			1.022		
AFP‐L3(ng/mL)						
>1.0	1	0.519–0.894	0.006	1	0.485–1.269	0.322
≤1.0	0.681			0.784		
GP73(ng/mL)						
>150	1	1.220–2.049	0.001	1	1.193–2.009	0.001
≤150	1.581			1.548		
ALB(g/L)						
<35	1	0.822–1.359	0.666			
≥35	1.057					
TBil(μmol/L)						
>19	1	1.038–1.732	0.025	1	0.877–1.544	0.294
≤19	1.341			1.164		
ALT(U/L)						
>40	1	1.041–1.780	0.024	1	1.005–1.724	0.046
≤40	1.361			1.316		

**TABLE 3:** Univariate analysis and multivariate analysis for OS

Variables	Univariate analysis			Multivariate analysis		
	HR	95% CI	*p*	HR	95% CI	*p*
Gender						
Male	1	0.153–1.918	0.342			
Female	0.541					
Age (y)						
>56	1	0.274–2.085	0.589			
≤56	0.756					
Tumor Size(cm)						
>3	1	0.096–0.762	0.013	1	0.109–0.906	0.032
≤3	0.271			0.315		
Metastasis						
No	1	0.362–4.553	0.699			
Yes	1.284					
Differentiation						
Low	1	0.050–2.897	0.351			
High	0.381					
Therapeutic Methods						
Resection	1					
Interventional therapy	1.103	0.141–8.615	0.926			
Cryoablation	1.193	0.165–8.632	0.861			
Radiofrequency ablation	1.249	0.164–9.501	0.830			
Comprehensive therapy	1.000	0.133–7.491	1.000			
AFP(ng/mL)						
>25	1	0.122–1.201	0.1			
≤25	0.382					
AFP‐L3(ng/mL)						
>1.0	1	0.185–1.827	0.353			
≤1.0	0.582					
GP73(ng/mL)						
>150	1	0.000–5.907	0.198			
≤150	0.033					
ALB(g/L)						
<35	1	0.035–0.692	0.014	1	0.028–0.570	0.007
≥35	0.156			0.127		
TBil(μmol/L)						
>19	1	0.060–0.754	0.016	1	0.108–1.584	0.197
≤19	0.213			0.413		
ALT(U/L)						
>40	1	0.093–0.794	0.017	1	0.079–0.710	0.01
≤40	0.271			0.237		

### 3.4 Subgroups analysis with serum markers for RFS

In order to obtain more in‐depth interpretations, we further carried out the subgroup analysis according to the tumor size and therapeutic methods in recurrent populations. We did not perform subgroup analysis in the survival population due to small sample size. We divided all recurrent records into two groups, and patients with tumor size less than 3cm were defined as early‐stage tumor or else were defined as advanced stage tumor. From Table 4, we can see that recurrent time with four markers (AFP, AFP‐L3, GP73, and ALT) also had significant difference in advanced stage tumor group (*p *< 0.05). However, in early‐stage tumor group, only GP73 and ALT still had significant difference.

**TABLE 4:** Subgroup analysis with tumor size and therapeutic methods in recurrent patients

Variables	Tumor Size				Therapeutic Methods									
	>3 cm		≤3 cm		Resection		Interventional therapy		Cryoablation		Radiofrequency ablation		Comprehensive therapy	
	Median	*p*	Median	*p*	Median	*p*	Median	*p*	Median	*p*	Median	*p*	Median	*p*
AFP(ng/mL)														
>25	8	0.005	10	0.433	8	0.397	9	0.016	7	0.019	9	0.347	10	0.372
≤25	13		12		5		17		13		12		9	
AFP‐L3(ng/mL)														
>1.0	8	0.001	9	0.268	9	0.400	9	0.008	7	0.009	8	0.570	10	0.327
≤1.0	16		13		11		20		15		24		9	
GP73(ng/mL)														
>150	13	0.004	13	0.023	14	0.269	14	0.012	13	0.006	12	0.908	9	0.569
≤150	8		9		8		8		7		9		11	
ALB(g/L)														
<35	10	0.861	11	0.547	7	0.601	12	0.384	9	0.591	12	0.713	3	0.048
≥35	9		12		10		12		8		14		11	
TBil(μmol/L)														
>19	11	0.205	12	0.048	7	0.112	13	0.322	8	0.755	29	0.008	11	0.504
≤19	9		9		10		11		9		6		9	
ALT(U/L)														
>40	12	0.022	11	0.205	9	0.125	16	0.134	10	0.589	9	0.658	10	0.270
≤40	8		11		8		10		7		12		7	

The results of subgroups analysis in therapeutic methods showed that all the serum markers had no significant differences in patients with surgical resection (*p *> 0.05). As regards interventional therapy and cryoablation, three tumor markers (AFP, AFP‐L3, and GP73) also had significant differences (*p *< 0.05). For radiofrequency ablation and comprehensive therapy, there was only one significant prognostic marker (*p *< 0.05), and TBil had significant difference in radiofrequency ablation (*p* = 0.008) and ALB in comprehensive therapy (*p* = 0.048).

## 4 DISCUSSION

Lots of attentions have been paid to the prognostic biomarkers for HCC in the past several years. New genetic detection technologies have been promising tools for potential biomarkers discovery. Cell‐free DNA, lncRNA, and mRNA expression also had important prognostic value for HCC according to the previous studies.^14^, ^15^, ^16^ However, these genetic biomarkers are still far from being applied in clinical diagnosis due to high cost and complex manufacturing processes. Traditional routine tests still play pivotal roles in the prediction of outcomes in HCC patients at present.^17^, ^18^, ^19^

In this study, we investigated the prognostic values of six serum markers in HCC populations after treatments based on RWD records. Three tumor biomarkers and three liver function tests were analyzed in RFS and OS, respectively. Wang NY et al^7^ evaluated prognostic values of these same three tumor biomarkers with HCC patients after radiofrequency ablation, and their results demonstrated that the short‐term (6 months) recurrence rate of AFP‐positive patients was obviously higher than that of AFP‐negative patients, but AFP‐L3 and GP73 levels were not associated with short‐term recurrence. However, our results in this study showed that all these three biomarkers were strong predictors of long‐term (60 months) recurrence in patients with HCC. Kim SH et al.^20^ obtained the similar results from HCC patients after liver transplantation. They also indicated that high level of AFP was a significant independent risk factor for recurrence, and the median time to recurrence was 10 months. Montalvá EM et al.^21^ followed up HCC patients after liver transplantation more than 60 months, and they found that the OS and RFS were both differed between groups when an AFP cutoff level of 1,000 ng/mL was used. We used 25ng/mL as cutoff level in this study, and RFS was also differed between groups, but OS was not (*p* = 0.085). AFP is still the most widely used tumor markers for HCC, and our present study and previous studies have confirmed its prognostic value in the recurrence of HCC. However, its prognostic value in survival time is still needed to be validated in the future.

GP73 is a resident Golgi‐specific membrane protein expresses by biliary epithelial cells in the normal liver. Our previous studies had reported that it is superior to AFP as an early diagnostic biomarker for HCC, and serum GP73 level was significantly decreased during the progression of HCC.^22^, ^23^ As a serological marker, GP73 has always been controversial. Some reports pointed out that GP73 should be an indicator of liver inflammation and fibrosis instead of a tumor marker.^24^, ^25^, ^26^, ^27^ Some research results showed that the expression of serum GP73 was significantly higher in the HCC patients compared with the liver fibrosis and cirrhosis patients.^28^, ^29^ However, there are few reports on the prognostic value of GP73 in HCC patients. In this study, we also found that GP73 was an outstanding prognostic biomarker for recurrent and survival prediction after treatment in HCC patients. Multivariate analysis also demonstrated that GP73 was an independent adverse prognostic factor for RFS prediction. Our findings showed that patients with higher GP73 experienced longer RFS and shorter OS. Except for GP73, ALT also was proven as an independent adverse prognostic factor for RFS in our study, and patients with higher ALT had longer RFS and shorter OS. In this study, ALT and ALB were identified as independent prognostic factors for OS. Higher ALT and lower ALB indicate that liver function is damaged severely, and it is the cause of high death rate in patients. However, for the reasons of higher ALT and TBil patients had longer RFS are still unclear.

We also investigated the RFS of six serum markers in different stages and therapeutic methods by subgroup analysis. In advanced stage HCC, six serum markers had similar performance with overall population, but in early stage, GP73 had more superiority than other markers. This result revealed that GP73 had better prognostic value than others, particularly in early‐stage HCC. Subgroup analysis in therapeutic methods also provided some new findings. Three tumor biomarkers only had significant RFS in interventional therapy and cryoablation, but they had not obvious values in RFS prediction in other therapeutic methods.

There are still some limitations need to be taken into account in this study. We used retrospective RWD for analysis; though RWD can reflect the more realistic values, the results will be affected by various confounding factors and produce lower credibility compared with trial data. Moreover, we enrolled 104 patients in OS analysis, but only 15 of them were verified dead by electronic medical records. This proportion is lower than the mortality rate of HCC reported in literatures, because the final outcomes in most records were not available due to ethical issues.

In conclusion, our study demonstrates that three tumor biomarkers, including AFP, AFP‐L3, and GP73, can be used as prognostic factors for predicting the recurrence of HCC, but liver function tests seem to have better survival prediction values. GP73 can act as a promising prognostic indicator for HCC, particularly in early‐stage HCC. However, larger‐scale prospective studies with multicenter are still needed to further verify our findings.

## CONFLICT OF INTEREST

The authors declare no competing financial interests.

## Data Availability

The data used to support the findings of this study are available from the corresponding author upon request.
